# Genetic Analyses, BSA-Seq, and Transcriptome Analyses Reveal Candidate Genes Controlling Leaf Plastochron in Rapeseed (*Brassica napus* L.)

**DOI:** 10.3390/plants14111719

**Published:** 2025-06-05

**Authors:** Mengfan Qin, Xiang Liu, Jia Song, Feixue Zhao, Yiji Shi, Yu Xu, Zhiting Guo, Tianye Zhang, Jiapeng Wu, Jinxiong Wang, Wu Li, Keqi Li, Shimeng Li, Zhen Huang, Aixia Xu

**Affiliations:** 1 College of Agronomy, Northwest A&F University, Yangling 712100, China; qinmf@nwafu.edu.cn (M.Q.); liux@nwafu.edu.cn (X.L.); s13563403318@163.com (J.S.); 17873555895@163.com (F.Z.); shiyj0718@nwafu.edu.cn (Y.S.); xuyu_xy@nwafu.edu.cn (Y.X.); 18003852705@163.com (Z.G.); giornozhang@nwafu.edu.cn (T.Z.); jiapengwuu@outlook.com (J.W.); likeqi1218@sina.com (K.L.); huang_zhen.8@163.com (Z.H.); 2 Crop Research Institute, Guangdong Academy of Agricultural Science, Guangzhou 510640, China; liwu@gdaas.cn; 3 Institute of Agricultural Sciences, Xizang Academy of Agriculture and Animal Husbandry Sciences, Lhasa 850000, China; wjxwang9619@163.com

**Keywords:** *Brassica napus* L., leaf plastochron, genetic analysis, BSA-seq, gene expression, candidate gene

## Abstract

The leaf plastochron serves as an indicator of the rate of leaf appearance, biomass accumulation, and branch number, while also impacting plant architecture and seed yield. However, research on the leaf plastochron of crops remains limited. In this study, 2116C exhibited a rapid leaf plastochron compared to ZH18 during both rosette and bud periods. There were significant positive correlations among the leaf plastochron and primary branch number of the F_2_ populations (*r* ranging from 0.395 to 0.635, *p* < 0.01). Genetic analyses over two years demonstrated that two equally dominant genes might govern the leaf plastochron. Through bulk segregant analysis sequencing (BSA-seq), three novel genomic intervals were identified on chromosomes A02 (9.04–9.48 Mb and 13.52–13.66 Mb) and A04 (19.84–20.14 Mb) of ZS11 and Darmor-*bzh* reference genomes. By gene functional annotations, single-nucleotide variation (SNV) analyses, transcriptome data from parents, genetic progeny, and natural accessions, we identified ten candidate genes within the intervals, including *FLOWERING LOCUS T*, *RGL1*, *MYB-like*, *CYP96A8*, *BLH3*, *NIT2*, *ASK6*, and three *CLAVATA3/ESR (CLE)-related* genes. These findings lay the molecular foundation for further exploration into the leaf plastochron and the implications in plastochron-related breeding in rapeseed.

## 1. Introduction

The leaf plastochron, defined as the temporal interval between the initiation of two successive leaf primordia at the shoot apical meristem (SAM), is a critical parameter in plant developmental studies [1,2]. The shorter the leaf plastochron indicates, the faster the rate of leaf initiation and leaf appearance. The final number of leaves depends upon the rate and pattern of leaf initiation during the growth phase [3,4]. During leaf axillary meristem development, axillary buds are regulated by diverse signals together to form into branches, e.g., polar transport of auxin, abscisic acid from buds, cytokinins from root tips, strigolactone, sugar signaling, and red light [5,6,7,8]. Consequently, the higher final leaf count corresponds to the more axillary meristems, potentially leading to enhanced branch formation. It is known that leaves and branches serve as key organs for photosynthesis and the architecture of rapeseed [8,9]. Thus, the leaf plastochron holds significance for improving biomass, plant architecture, and photosynthesis in crops.

Despite the importance of leaf plastochron pattern in the plant architecture, little is known about the genetic regulation and molecular mechanisms of the leaf plastochron. Leaf initiation originates from shoot apical meristems (SAMs), whose organization and function were maintained by the conserved WUSCHEL-CLAVATA3 (WUS-CLV3) feedback loop [10]. In rice (*Oryza sativa* L.), mutations in *PLASTOCHRON1* (*PLA1*) and *PLA2* accelerate leaf initiation without altering phyllotaxy [1,2,11]. *PLA1* regulates the leaf plastochron by modulating meristem developmental timing, and *PLA2* may mediate non-cell-autonomous signals from immature leaves to suppress new leaf initiation in the shoot apical meristem. Both of them act as the downstream of the GA signal transduction pathway [12], and PLA1 also regulates leaf inclination through the brassinolide pathway [13]. In maize (*Zea mays*), *ZmPLA1* maintains the dividing of cells in a proliferative and undifferentiated state for a longer time, thereby stimulating the duration of leaf elongation, which could increase organ growth, seedling vigor, biomass, and seed yield [14]. In addition, the leaf plastochron will also vary according to the light and cumulative temperature, which influences the duration of nutrient growth, developmental rate, total leaf number, biomass, and seed yield of plants [15,16,17]. Although the mechanisms underlying leaf plastochron regulation remain to be elucidated in dicot crops such as rapeseed, the identified regulatory links to SAMs and hormone signaling provide a directional framework for future exploration.

Rapeseed (*Brassica napus* L., AACC, 2n = 38) is a globally significant oil crop with extensive cultivation areas and is one of the four largest oil crops in the world. It not only provides edible oil for humans and protein-rich feed for animals, but also provides a series of novel vegetables, such as sprouts, leaves, and shoots [18,19,20]. The rapid-plastochron varieties could grow fast with more leaves, branches, and shoots, which have certain application value for vegetable, edible oil, green manure, and oil-vegetable-dual-purpose rapeseed. Therefore, it is meaningful to explore and identify rapid plastochron germplasm and genetic resources to facilitate the utilization and improvement of leaf plastochron-related traits. In this study, two rapeseed inbred lines with various plant plastochrons were used for progeny population construction. We performed major genes plus polygenes inheritance analyses for plastochron-related traits across two years among F_2_ populations and constructed bulk pools using extreme F_2_ individuals for Bulked Segregant Analysis (BSA) sequencing to identify candidate genomic intervals. Candidate genes were then predicted by gene sequence variation and expression information within the intervals. These results will help to expand our knowledge of the genetic mechanism of leaf plastochron and provide a theoretical basis for plastochron-related breeding and molecular biology in rapeseed.

## 2. Results

### 2.1. Investigation of Plastochron for the Parents

**Figure 1:**
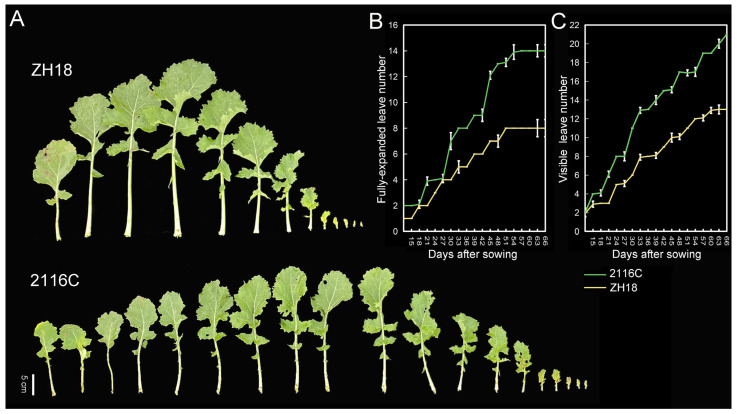
Leaf appearance rate at the rosette stage of ZH18 and 2116C. (**A**) Final leaf number of ZH18 and 2116C on the 69th day after sowing; (**B**) fully expanded leaves; and (**C**) visible leaves during the rosette stage. The x-axis is the number of days after sowing, and the y-axis is the leaf number. The green and yellow lines indicate 2116C and ZH18, respectively.

To investigate the leaf plastochron pattern in rapeseed, the rates of leaf appearance of ZH18 (slow-plastochron line) and 2116C (rapid-plastochron line) were continuously counted before the wintering period. During the pre-winter growth process, there were two peaks of leaf plastochron in both lines, and the leaf appearance rate showed the characteristics of ‘slow-rapid’ alternation. ZH18 had the fastest leaf plastochron in the 3rd–4th and 7th–8th leaves, while 2116C had the fastest in the 5th–8th and the 10th–13th leaves. The final number of fully expanded leaves of ZH18 and 2116C were 8 leaves and 14 leaves, while their visible leaves were 13 leaves and 20 leaves, respectively (Figure 1). The average leaf appearance rate was 3.2 days/leaf in ZH18 and 5.1 days/leaf in 2116C. 2116C consistently had significantly higher leaf numbers and leaf plastochron than ZH18 at the rosette stage (Figure 1), which indicated that there was likely to be a certain mutation consistently affecting the leaf appearance and plastochron all the time.

### 2.2. Phenotype Investigation for the Parents and Genetic Population

**Figure 2:**
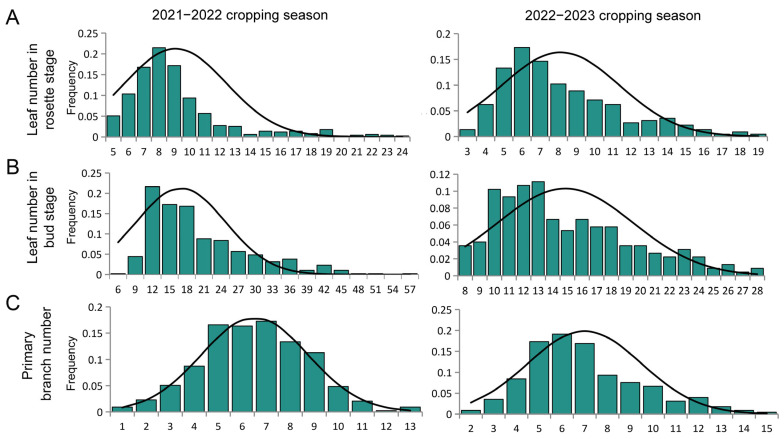
Statistical analyses of leaf plastochron-related traits among the F_2_ populations. Frequency distribution of (**A**) leaf number in the rosette stage, (**B**) leaf number in the bud stage, and (**C**) primary branch number across 2021–2022 and 2022–2023 cropping seasons.

To conveniently study the genetic characteristics of the leaf plastochron, we subsequently used the leaf number at the same number of days after sowing to represent the leaf plastochron of the individual. From the phenotypic survey results of the 2021–2022 and 2022–2023 cropping seasons (Table S1), the mean values of expanded leaf number at the rosette stage were 5.3 and 7.5 in ZH18, 12.2 and 13.6 in 2116C, 9.6 and 10.1 in F_1_ plants, and 8.1 and 9.1 in F_2_ population, respectively. During the bud stage, the mean values of the number of leaves were 7.6 and 10.6 in ZH18, 23.1 and 18.4 for 2116C, 14.9 and 15.5 in the F_1_ plants, and 17 and 14.9 in the F_2_ population, respectively. The number of leaves in F_1_ was intermediate between the two parents, and the leaf number of the F_2_ population showed a unimodal distribution (Figure 2). Therefore, the leaf plastochron at the rosette-bud stages of rapeseed has a quantitative trait genetic character. The correlation analysis showed that there were highly significant correlations among leaf plastochron at the rosette stage and the bud stage (*p* < 0.01) with the primary branch number (*p* < 0.01) (Table S2). These results indicated that there might be an intrinsic positive correlation between the leaf plastochron and the primary branch number.

### 2.3. Genetic Analysis of Leaf Plastochron in Rapeseed

To predict the mixed major genes plus polygenes inheritance of leaf plastochron in rapeseed, we utilized the leaf number of ZH18, 2116C, F_1_, and F_2_ in the 2021–2022 and 2022–2023 cropping seasons for genetic model prediction. Based on the principle of minimum Akaike’s information criterion (AIC) value, the alternative models for leaf plastochron at the rosette stage were one major gene with equal additive-dominance (1MG-EAD) and two major genes with equal additive-dominance (2MG-EAD), and those for leaf plastochron at the bud stage were 2MG-EAD and two major genes plus polygenes with completely dominance and additive-dominance (MX2-CD-AD) across the two cropping seasons (Table 1). Similarly, the most appropriate genetic models for primary branch numbers were 2MG-EA and 2MG-EAD. In the fitness test, the models with more counts of significant *p*-values (*p* < 0.05) were selected as the optimal genetic models (Table S3). Taken together, the 2MG-EAD genetic model was the most appropriate model for leaf plastochron after the fitness test.

Using the 2MG-EAD model, we predicted the genetic parameters for leaf-related traits. The additive effects of major genes were consistently negative, with heritability exceeding 86.4% (Table S4). It was evident that leaf plastochron was mainly controlled by two equally dominant effect genes with high heritability and negative additive-dominant effects. These results could provide cues for the exploration of leaf plastochron in rapeseed.

### 2.4. Primary QTL Identification Based on BSA Analyses

**Figure 3:**
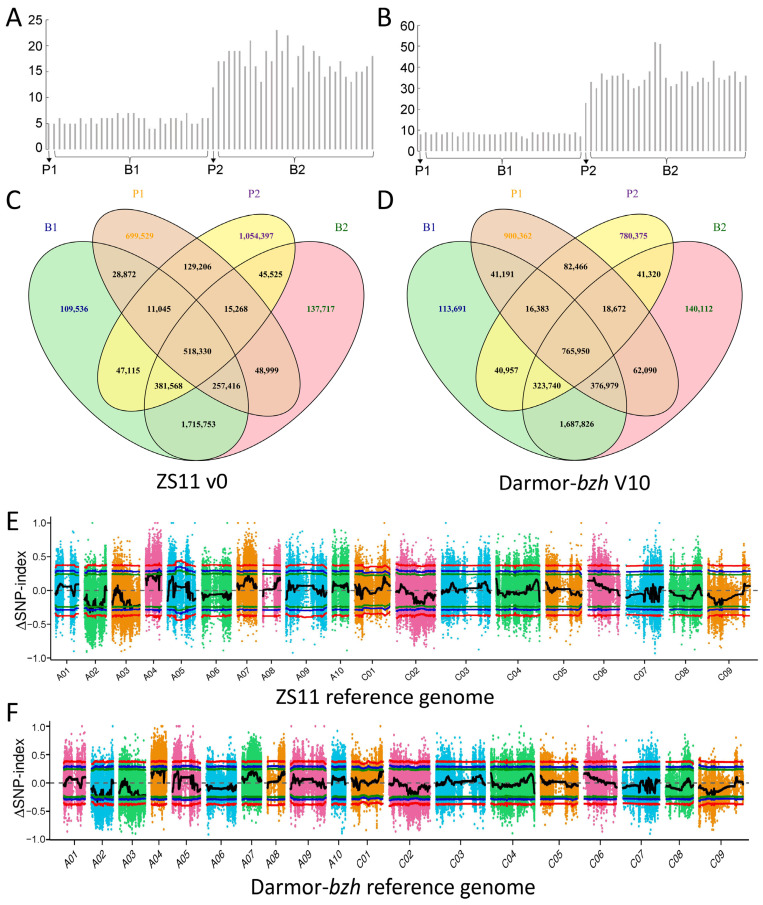
Leaf plastochron QTL identification based on BSA-seq. Leaf number in (**A**) the rosette stage and (**B**) the bud stage; Venn diagrams of SNPs identified in the samples aligned to (**C**) ZS11 v0 and (**D**) Darmor-bzh reference genome; and ΔSNP-index based on (**E**) the ZS11 v0 and (**F**) the Darmor-*bzh* V10 reference genome. The red, blue, and green lines represent confidence thresholds of 0.99, 0.95, and 0.90, respectively. Different colors are used to distinguish the axes representing the different chromosomes.

To gain further access to the genome intervals controlling leaf plastochron, BSA sequencing for the parents and F_2_ population was approached. During the 2021–2022 cropping season, the two parents, along with two bulked pools B1 (slow plastochron bulk pool with 30 plants from 600 F_2_ individuals) and B2 (rapid-plastochron bulk pool with 30 plants from 600 F_2_ individuals) were selected for whole-genome resequencing (Figure 3). The number of leaves at the rosette stage and the bud stage was counted (Figure 2A,B). The reference genome alignment and read coverage statistics for resequencing of four samples were stated, and sequencing coverage at least 1X was >89.9% for the parents and F_2_ bulked pools (Table S5).

For the ZS11 v0 genome assembly, alignment of the reads from ZH18 and 2116C to the reference genome revealed a total of 2,264,093 and 2,069,863 SNPs, respectively. There were 3,366,717 and 3,416,689 SNPs determined from the B1 and B2 bulked pools, and 51,833 SNPs were shared among P1, P2, B1, and B2 (Figure 3C). In addition, 610,689, 583,140, 901,648, and 610,689 small InDels were detected from ZH18, 2116C, B1, and B2, respectively (Figure S1 and Table S5). Twenty genomic regions with a total length of 0.89 Mb were identified based on SNPs with a confidence interval of 95 on chromosomes A02, A03, and A04 (Figure 3E and Table S6). Twenty-eight regions on chromosomes A02 and A04 with a confidence interval of only 90 were obtained from 1,073,929 small InDels (Figure S2 and Table S6).

For the Darmor-*bzh* V10 genome assembly, a total of 765,950 SNPs and 207,694 InDels were shared among P1, P2, B1, and B2 (Figure 3D and Figure S1). A total of 28 genomic regions associated with SNPs were identified with a confidence interval of 95 on chromosomes A02 and A04 (Figure 3F and Table S7), while 17 regions for InDels with a confidence interval of only 90 were on chromosomes A02, A03, and A04 (Figure S3 and Table S7). Overall, the QTL outcomes of SNPs were preferred due to genetic density and the higher confidence interval, and 107 and 113 genes were located in the intervals from ZS11 and Darmor-*bzh*, respectively (Table 2).

The intervals between ZS11 and Darmor-*bzh* showed high genome collinearity, and 90 genes of ZS11 have more than 30% protein identity with that of Darmor-*bzh* (Figure S4 and Table S8), suggesting that the QTL intervals were conserved in rapeseed. Taken together, the candidate intervals controlling plant plastochron might be located in A02:9.04−9.48 Mb (labeled as qPLA.A02-1), A02:13.52−13.66 Mb (labeled as qPLA.A02-2), and A04:19.84−20.14 Mb (labeled as qPLA.A04) of the ZS11 genome.

### 2.5. Gene Annotations and Sequence Variants Analyses in Candidate Genes

**Figure 4:**
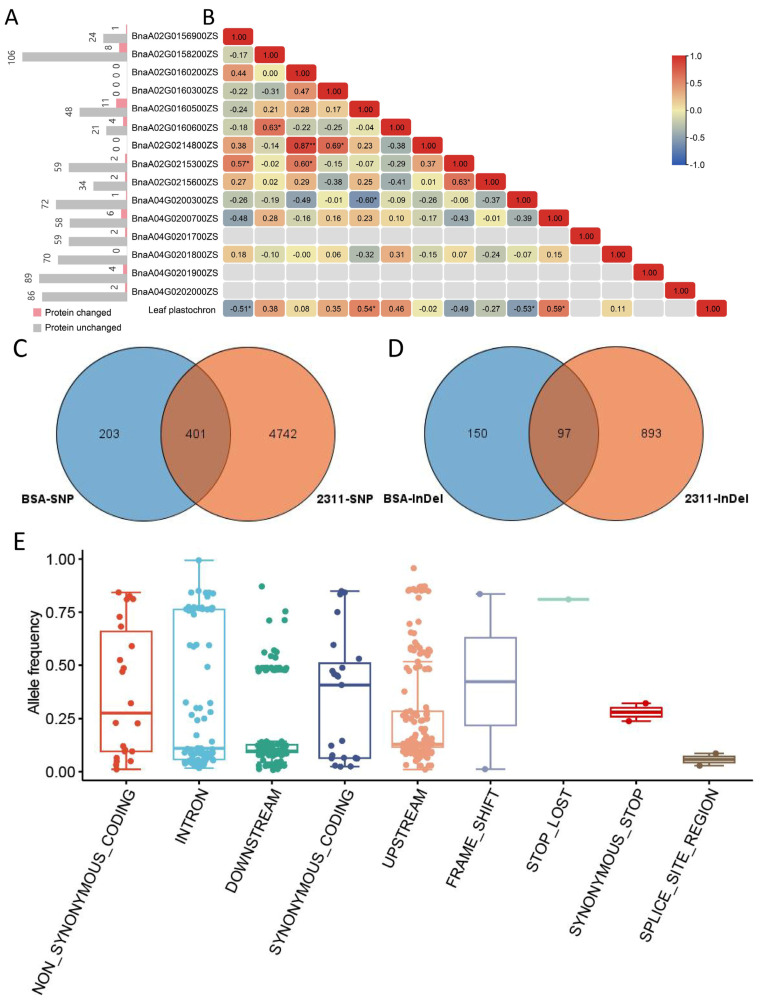
SNP/InDel variation and gene expression analyses of candidate genes. (**A**) Number of SNP/InDel in candidate genes leading to protein sequence change; (**B**) heatmap of correlation between candidate gene expression and leaf plastochron; The asterisk * represents a significance *p* < 0.05 and ** represents a significance *p* < 0.01. (**C**) SNP and (**D**) InDel Venn plots from BSA versus that from the 2311 accessions in BnIR database; and (**E**) allele frequency distribution of the common variants among the 2311 accessions.

Within the three QTL intervals, a total of 90 genome genes were identified in the ZS11 genome (Table 2). Among these genes, 15 genes might be the causal genes that annotated as *FLOWERING LOCUS T* (*BnaA02G0156900ZS*), DELLA protein (*BnaA02G0158200ZS* and *BnaA02G0160500ZS*), Senescence-associated protein (*BnaA02G0160200ZS*), *ACCELERATED CELL DEATH 6* (*BnaA02G0160300ZS*), *MYB-like* (*BnaA02G0160600ZS*), bifunctional nuclease 1 (*BnaA02G0214800ZS*), *CYP96A8* (*BnaA02G0215600ZS*), *BLH3* (*BnaA02G0215300ZS*), *Nitrilase 2* (*BnaA04G0200300ZS*), GSK3-like protein kinase (*BnaA04G0200700ZS*), G-protein coupled receptor (*BnaA04G0201800ZS*), and CLAVATA3/ESR (CLE)-related protein (*BnaA04G0201700ZS*, *BnaA04G0201900ZS*, and *BnaA04G0202000ZS*). According to SNP and InDel annotations, there were 604 and 247 variants identified among 12 of the 15 genes, including 312 in upstream, 325 in downstream, 142 in intron, 1 in 5’ UTR, 1 in 3’ UTR, 29 leading to synonymous coding, and 41 leading to non-synonymous coding (Figure 4A and Table S9). *BnaA04G0201700ZS* and *BnaA04G0201800ZS* only showed 143 and 70 variants in upstream, downstream, and synonymous coding.

Variation information of 2311 rapeseed accessions extracted 5143 SNPs and 990 small InDels from the 15 genes mentioned above, and 203 SNPs and 150 InDels from BSA sequencing were not detected in the database (Figure 4C,D), which were unique to the variations between the parents in this study. Among the parental-specific variants, they were distributed over 12 genes (Table S10), resulting in four altered protein sequences of them (*BnaA02G0215300ZS*, *BnaA02G0160600ZS*, *BnaA04G0200700ZS*, and *BnaA04G0201900ZS*). Among the 498 common variants, 27 variants were distributed in nine genes (*BnaA02G0156900ZS*, *BnaA02G0158200ZS*, *BnaA02G0160500ZS*, *BnaA02G0160600ZS*, *BnaA02G0215600ZS*, *BnaA04G0200300ZS*, *BnaA04G0200700ZS*, *BnaA04G0201900ZS*, and *BnaA04G0202000ZS*) and caused alterations of the encoded proteins (Table S10). The allele frequency of these variants ranged between 0.01 and 0.85 (Figure 4E), and those with low allele frequency variants were more likely to be the candidate loci.

### 2.6. Gene Expression Analyses in Candidate Genes

Given that the results of the phenotypic analyses suggest that plant plastochron might be controlled by continuous regulation, the candidate genes should be consistently expressed, whether at the stem tip or in leaf-associated tissues. For genes with protein sequence alterations, the expression of candidate genes may not correlate with leaf plastochron. For genes with unaltered protein sequences, the expression should correlate with leaf plastochron, and there were SNPs/InDel in their upstream, downstream, or UTR regions.

The principal components analysis (PCA) based on FPKM of the 15 candidate genes showed that the 12 RNA-seq samples were effectively divided into slow-plastochron and rapid-plastochron groups (Figure S5). For the gene expression among the parents and F_2:3_ families, only four genes (*BnaA02G0158200ZS*, *BnaA04G0201700ZS*, *BnaA04G0201900ZS*, and *BnaA04G0202000ZS*) did not show detected expression abundances, and the other genes showed different expression trends (Figure S6). The expressions of *BnaA02G0160500ZS* and *BnaA04G0200700ZS* displayed significantly positive correlations to leaf plastochron (*p* < 0.05, *r* = 0.54 and 0.59, respectively), while *BnaA02G0156900ZS* and *BnaA04G0200300ZS* showed negative correlations to leaf plastochron (*p* < 0.05, *r* = −0.51 and −0.53, respectively) (Figure 4B). In the public gene expression data, three genes (*BnaA04G0201700ZS*, *BnaA04G0201900ZS*, and *BnaA04G0202000ZS*) did not express, and three genes (*BnaA02G0158200ZS*, *BnaA02G0160600ZS*, and *BnaA04G0201800ZS*) showed low expression in all of these tissues (Table S11). *BnaA02G0156900ZS* was not expressed in cotyledons and roots but was highly expressed in different-period leaves (Table S11). In addition, we observed that there were three tandem repeats of *CLE* genes within the qPLA.A04 interval, and although they were not detected to be expressed in this study, since *WUS-CLV* was an essential feedback regulatory loop for maintaining the stability of the shoot apical meristem, we considered them as potential candidate genes as well.

Taken together, we regarded *BnaA02G0156900ZS* (*FT*), *BnaA02G0160500ZS* (*RGL1*), and *BnaA02G0160600ZS* (*MYB-like*) as potential candidate genes for qPLA.A02-1, *BnaA02G0215600ZS* (*CYP96A8*) and *BnaA02G0215300ZS* (*BLH3*) for qPLA.A02-2, and *BnaA04G0200300ZS* (*NIT2*), *BnaA04G0200700ZS* (*ASK6*), *BnaA04G0201700ZS* (*CLE4*), *BnaA04G0201900ZS* (*CLE6*), and *BnaA04G0202000ZS* (*CLE7*) for qPLA.A04 (Table 3).

## 3. Discussion

**Figure 5:**
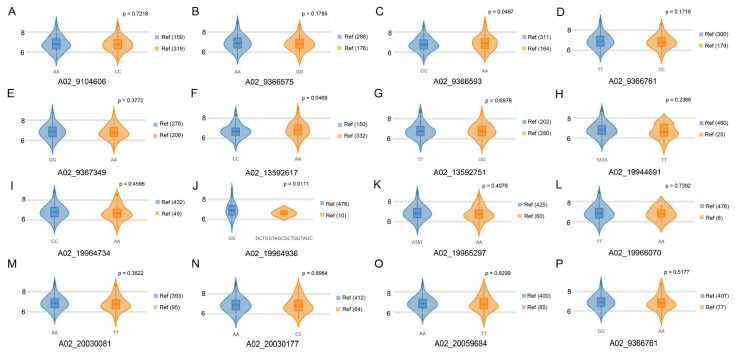
The relationships of branch numbers for 16 SNVs shared with natural accessions. (**A**–**P**) Violin diagrams of branch numbers of different genotypes. The individual numbers of the genotype are listed in the braces. The *p*-value was calculated by *t*-test.

Leaves are essential photosynthetic organs, and the leaf plastochron can affect the number of leaves, number of branches, biomass, and yield directly or indirectly, which is an important indicator of plant biology. In rice and wheat, the shorter the leaf plastochron, the less time tillers can be produced, which is important for yield and uniformity in grains [21]. In this study, significant positive correlations were found among the leaf plastochron, primary branch number, biomass, and seed yields (Table S2). Branches originated from axillary buds, and the leaves in the main stem, along with environmental factors, affected the number of branches and plant morphology [8,22], and multiple studies have reported positive correlations between branch number and biomass/seed yield [23,24,25]. These results are consistent with previous studies on the relationship between branch number and yield [26,27,28], i.e., the more branches, the higher the individuals’ biomass and seed yield. A branch number QTL was previously mapped via GWAS to a 22.0 Mb position on chromosome A03 [23], near which a QTL was also identified on the ZS11 v0 genome in this study (Table S7). Some allelic SNPs within the candidate genes exhibited significant effects on branch number (Figure 5), suggesting a potential intrinsic linkage among leaf plastochron, branch development, and biomass/seed yield. However, additional approaches are required to validate whether the observed associations represent causal relationships, genetic linkage, or coincidental occurrences.

To date, there are no genetic studies reported on leaf plastochron in rapeseed, and three novel QTL intervals were identified in this study. Notably, among the 10 leaf plastochron related candidate genes investigated in this study (including *FLOWERING LOCUS T, RGL1, MYB-like, CYP96A8, NIT2, ASK6,* and *CLE4/6/7*), 16 single-nucleotide variations (SNVs) were identified in natural populations. Of these, 3 allelic SNVs exhibited significant associations with branch number (Figure 5), while 13 SNVs showed correlations with flowering time (Figure S7), with allele frequencies ranging from 0.01 to 0.72. These results suggest that these candidate genes may participate in the regulation of both branch number and flowering time, extending their functional roles beyond leaf plastochron modulation. Consequently, superior haplotypes or genetic engineering targeting these leaf plastochron-related genes could be strategically employed in breeding programs to optimize plant architecture, early flowering, and yield-related traits, even breeding novel vegetables.

Three candidate genes, *BnaA02G0156900ZS* (*FT*), *BnaA02G0160500ZS* (*RGL1*), and *BnaA02G0160600ZS* (MYB-like), were identified in the qPLA.A02-1 interval. *BnaA02G0156900ZS* encoded an FT mobile signaling protein, whose mRNA was expressed in the leaves and responded to long-day conditions, but the protein was required by the shoot apical meristem [29,30]. The *ft-10* mutant had 36 leaves, while wild-type plants developed 15 rosette leaves at flowering [31]. There was one SNP that caused the protein change Ile49Leu located in the phosphatidyl ethanolamine-binding protein (PEBP) domain, which might be the functional site of *BnaA02G0156900ZS*. *BnaA02G0160500ZS* (*RGL1*), encoding a DELLA protein, negatively regulated GA responses and restrained the cell proliferation and expansion that drove plant growth [32,33]. There was no significant difference in gene expression between slow-plastochron lines and rapid-plastochron lines, but one InDel led to a frameshift, one SNP stop loss, and nine SNP non-synonymous coding (Table S9). Compared with the wild type, leaf senescence occurred earlier in the mutant *rgl1-1,* whose *DELLA* repression was removed, and overexpression of *RGL1* resulted in significantly increased leaf longevity in age-triggered senescence [34,35]. *BnaA02G0160600ZS* encoded a putative MYB domain containing a transcription factor that participated in several developmental processes. *R2R3-Myb* genes controlled a very early step of axillary meristem initiation, which impacted the axillary meristem formation [36], and overexpression of *Cymbidium CcMYB24* increased the number of leaves in *Arabidopsis* [37], suggesting that *MYB* members also functioned in the establishment of leaf primordia and branches.

In the qPLA.A02-1 interval, *BnaA02G0215300ZS* encoded a BEL1-like homeodomain protein, which was found ubiquitously in plants and played important roles in regulating the meristem. In *Arabidopsis thaliana*, three BELL-family proteins, ATH1, PNY, and PNF, were combined to keep the initiation and maintenance of the SAM [38]. BLH proteins interact with *KNOX* genes to regulate early internode patterning events, while PNY-STM heterodimers are critical for SAM function [38,39]. *BnaA02G0215600ZS*, a cytochrome P450 family member, had two SNP missense variants between the parents that caused protein changes Phe10Leu and Leu55Arg. *PLA1*, encoding cytochrome P450 CYP78A11, acts as a developmental timekeeper for leaf initiation in rice, orchestrating leaf number, tiller number, primary branch development, leaf inclination, leaf elongation, and leaf maturation through GA and brassinosteroid signaling pathways [1,2,11,12,13,40]. Deleting the P450 family member *MAX1* led to more branches via the strigolactone synthesis pathway, and overexpression of *BnaMAX1* could restore the multi-branch phenotype [27,41].

*BnaA04G0200300ZS* was homologous with *AtNIT2*, and mutants of *AtNIT2* reduced sensitivity to 3-Indoleacetonitrile (IAN), increased sensitivity to IAA, showing more rosette leaves in short days in *Arabidopsis* [42,43]. The gene *BnaA04G0200700ZS* encoded the apoptosis signal-regulating kinase (ASK) protein, also known as *BRZ-INSENSITIVE-LONG HYPOCOTYL 1* (*BIL1*), *BRASSINAZOLE-RESISTANT 1* (*BZR1*), and *BRASSINOSTEROID INSENSITIVE 2* (*BIN2*). Phosphorylation of BRI1 EMS SUPPRESSOR 1 (BES1) and BZR1 by BIN2 facilitated their interaction with 14-3-3 proteins, leading to their translocation from the nucleus to the cytoplasm, thereby impeding their nuclear activities as transcription factors in *Arabidopsis* [44,45]. BIL1/AtSK23 also phosphorylated *MONOPTEROS/AUXIN RESPONSE FACTOR 5* at threonine 163 and serine 647, which could integrate *PHLOEM INTERCALATED WITH XYLEM* and cytokinin signaling in secondary growth [46]. CLE peptides played a role in controlling the growth and maturation of different tissues in *Arabidopsis*, including the apical and vascular meristems. Notably, CLV3 and CLE40 were recognized as CLE peptides that modulated the WUS-CLV feedback loop within SAM [47]. However, three members of CLE peptides were identified in qPLA.A04 without expression in the shoot tips, implying that they may not participate in leaf plastochron controlling.

## 4. Materials and Methods

### 4.1. Plant Plantation and Phenotype Collection

The winter rapeseed inbred lines ZH18 (slow-plastochron line) and 2116C (rapid-plastochron line) were provided by the rapeseed germplasm resource lab of Northwest A&F University, from which F_1_ and F_2_ populations were constructed. ZH18, 2116C, F_1_, and F_2_ were planted in the Caoxinzhuang experimental base (34°30′ N, 108°09′ E) of Northwest A&F University in Yangling during the 2021–2022 and 2022–2023 cropping seasons (experimental design was displayed in Figure S8). The F_2_ populations contained 600 and 225 individuals in the two cropping seasons, respectively. The plants during the 2021–2022 season were used for the analyses of the leaf plastochron pattern and inheritance, and those during the 2022–2023 cropping season were utilized for studying the inheritance aspects of leaf plastochron. Row length, row spacing, and plant spacing were set to 2 m, 35 cm, and 10 cm, respectively. Drill sowing was used, and thinning out of seedlings was performed before the two-leaf stage. Other field management was kept in alignment with local agronomic protocols.

Leaf plastochron variation patterns in the two parental lines were investigated every 3 days starting from 15 days after sowing, with one row examined during each observation. The numbers of fully expanded leaves and visible leaves were recorded, with five representative plants assessed per survey and two replicates. The F_2_ plants were investigated one by one, and border row plants were excluded from evaluations to avoid marginal effects. The leaf numbers were investigated in the rosette stage and the bud stage, representing the leaf plastochron of the plants. The effective primary branch numbers were in the final flowering period. Biomass and seed yields were obtained from 40 F_2_ plants exhibiting extreme leaf plastochron phenotypes, including 20 individuals each from fast-plastochron and slow-plastochron plants. The mature aboveground biomass was placed into net bags, and both the sun-dried plant weight and seed weight were measured.

The extreme 10 F_2:3_ families were selected from the F_2_ population during the 2021–2022 cropping season, and 30 individuals for each family were planted in the chamber with condition settings (24 °C day/16 °C night temperatures and 14 h/10 h light/dark cycle). Given the trait segregation in F_2:3_ families, plant individuals with higher leaf numbers were selected from fast-plastochron families, and low-leaf-number lines were chosen from slow-plastochron families. The stem apexes of five plants were selected from each F_2:3_ family. A total of 12 samples, including two parental lines, were used for RNA sequencing.

### 4.2. Segregation Analysis for Leaf Plastochron-Related Traits

During the 2021–2022 and 2022–2023 cropping seasons, ZH18, 2116C, F_1_, and F_2_ populations were used for segregation analysis by using G4F2 (P_1_, P_2_, F_1_, and F_2_) of the SEA v2.0.1 R package [48]. According to the AIC minimum criterion, 4 models with the minimum AIC values were selected as candidate models from the 24 models [48]. Then, the fitness test was conducted for each candidate model separately, and the module with the minimum *p*-value (*p* < 0.05) was taken into consideration as the best model [48].

### 4.3. DNA Isolation and BSA-Sequencing

The two parents (ZH18 and 2116C) and two extremely bulked pools (B1 and B2) from the 600 F_2_ individuals were used for BSA-seq. The F_2_ bulked pools were constructed by the top 30 slow-/rapid-plastochron individuals, respectively. Genomic DNA was isolated from the fresh leaves using the cetyl trimethyl ammonium bromide (CTAB) protocol [49]. DNA was randomly broken into 350 bp fragments by ultrasonic waves, and the DNA fragments were used for sequencing library construction and Illumina HiSeq sequencing. Whole-genome re-sequencing in 20X and 30X coverage was performed on the parents and the bulked pools, respectively. Whole-genome sequences were generated using paired-end sequencing on the Illumina Hiseq platform. BSA (bulked segregant analysis) sequencing library preparation and sequencing were conducted at Biomarker Technologies Corporation (Beijing, China).

Raw reads were filtered by fastp v0.21.0 [50], then clean reads were obtained for subsequent analysis. The reads were mapped to the reference genome ZS11 v0 and Darmor-*bzh* V10 using Burrows-Wheeler-Alignment software (BWA) v2.2 [51,52]. Duplicate reads were sorted and removed with SAMtools v1.9 [53]. SNP and small InDel variant detections were performed by the HaplotypeCaller algorithm of GATK v3.8 [54], and the variants were annotated using SnpEff v3.6c software with the default parameters [55].

### 4.4. SNP-Index Calculation and QTL Detection

Before association analysis, variants were filtered with the criteria: filter out variants with (I) multiple genotypes, (II) with coverage less than 4X in the bulked pools, (III) that were homozygous and consistent between the two bulked pools or between the two parents, and (IV) that were inconsistent between the pool and parent with the same trait. Calculating the SNP-index is an association analysis method to find the significant differences in genotype frequency between the pools, and the ΔSNP(InDel)-index was obtained [56]. To determine and obtain the association threshold, the DISTANCE/SNPNUM method was used, and the regions higher than the threshold were considered trait-related candidate regions [57].

### 4.5. RNA Isolation and RNA-Seq Analyses

The stem apexes of ZH18, 2116C, and extremely F_2:3_ families were sampled for RNA isolation. The RNA isolation protocol followed the manufacturer’s instructions for the RNAprep kit, and the first strand of cDNA synthesis was performed using the FastKing Premix Kit (Tiangen, Beijing, China). The cDNA library preparations and sequencing experiments were conducted by the sequencing cooperation of Grandomics company (Wuhan, China), and the libraries were sequenced on an MGI platform. The fastp v0.21.0 was employed to obtain clean data from raw data [50], and the clean reads were mapped to the reference genome ZS11 v0 using Hisat 2.0 with default parameters [51]. Novel transcripts were identified by the Stringtie v2.1.7. Gene expression levels were determined using the fragments per kilobase of exon per million fragments mapped (FPKM).

### 4.6. Gene Prediction in the Mapping Interval

All genes within the targeted mapping intervals were identified from the *Brassica napus* ZS11 v0 genome [51]. Gene annotations were performed by BLAST in Nr (NCBI non-redundant protein sequences, https://ftp.ncbi.nlm.nih.gov/blast/db/, accessed on 20 April 2024.), Pfam (Protein family, http://pfam-legacy.xfam.org/https://ftp.ncbi.nlm.nih.gov/blast/db/, accessed on 27 January 2025.), KOG/COG (Clusters of Orthologous Groups of proteins, https://www.ncbi.nlm.nih.gov/research/cog/, accessed on 28 January 2024.), Swiss-Prot (https://www.expasy.org/resources/UniProtKB-swiss-prot, accessed on 20 January 2024.), KO (KEGG Ortholog database, https://www.genome.jp/kegg/ko.html, accessed on 20 January 2024.), and GO (Gene Ontology, https://geneontology.org/, accessed on 20 January 2024.) databases.

### 4.7. Candidate Gene Expression and Variants in the Public Database

The TPM value of target genes in the public transcriptome was extracted from the BnIR database [58], including cotyledon, vegetative rosette, leaves, root, seed, silique, and stem peel. The SNP and InDel information of 2311 rapeseed accessions were obtained from the BnIR database as well [58]. The single-nucleotide variations (SNVs), flowering time, and branch number of natural accessions were from the public data [59,60].

### 4.8. Data Analyses and Visualization

The phenotypic data were analyzed and visualized by Excel 2016 and R 4.0.2 (https://www.r-project.org/, accessed on 20 January 2024.). Significant differences were evaluated using the *t*-test method, and the *p*-value < 0.05 was considered to be significant. Data were means ± SD (standard deviation) of the biological samples. The visualizations of the BSA results were performed using BMKCloud v2024 (www.biocloud.net, accessed on 28 January 2024.).

## 5. Conclusions

In the present study, morphology, genetics, genomics, and transcriptomics were integrated to analyze the genetic mechanisms of leaf plastochron in rapeseed. Leaf expansion of the rapid-plastochron line 2116C was consistently faster than that of the slow-plastochron line ZH18, and the leaf plastochron showed a significant positive correlation with primary branch number. Genetic analyses demonstrated that leaf plastochron was mainly controlled by two equally dominant effect genes (2MG-EAD) via segregation analysis, and three novel associated intervals were detected in chromosome A02 (9.04–9.48 Mb and 13.52–13.66 Mb) and A04 (19.84–20.14 Mb) by BSA sequencing. A total of 10 candidate genes were identified in the intervals, including *FT* (*BnaA02G0156900ZS*), *RGL1* (*BnaA02G0160500ZS*), *MYB-like* (*BnaA02G0160600ZS*), *CYP96A8* (*BnaA02G0215600ZS*), *BLH3* (*BnaA02G0215300ZS*), *NIT2* (*BnaA04G0200300ZS*), *ASK6* (*BnaA04G0200700ZS*), and *CLEs* (*BnaA04G0201700ZS*, *BnaA04G0201900ZS*, and *BnaA04G0202000ZS*). These results could provide a foundation for exploring the leaf plastochron of 2116C and plastochron-related breeding in rapeseed.

## Figures and Tables

**Table 1:** Candidate genetic models for leaf plastochron-related traits.

Trait	Model of (2021–2022)	AIC Value	Log Max Likelihood Value	Model (2022–2023)	AIC Value	Log Max Likelihood Value
Leaf number in the rosette stage	1MG-EAD	2516.879	−1253.44	2MG-EAD	1210.203	−601.102
	1MG-AD	2518.637	−1253.319	1MG-AD	1212.56	−600.28
	2MG-EAD	2525.118	−1258.559	MX2-CD-AD	1217.865	−605.933
	2MG-EA	2534.13	−1263.065	2MG-A	1218.415	−604.208
	MX2-ADI-ADI	2546.923	−1261.461	1MG-EAD	1218.642	−604.321
Leaf number in the bud stage	2MG-EAD	2936.697	−1464.349	MX2-CD-AD	1352.254	−673.127
	2MG-EA	2942.665	−1467.333	MX2-EAD-AD	1352.505	−674.253
	1MG-AD	2946.55	−1467.275	2MG-EAD	1353.224	−672.612
	MX2-ADI-AD	2969.929	−1475.965	MX2-AD-AD	1354.104	−672.052
	MX2-ADI-ADI	2975.617	−1475.808	1MG-AD	1357.429	−672.715
Primary branch number	2MG-EA	1959.893	−975.9467	2MG-EAD	1089.852	−540.926
	2MG-EAD	1962.112	−977.0562	1MG-EAD	1091.632	−540.816
	1MG-EAD	1964.117	−977.0583	1MG-AD	1093.476	−540.738
	1MG-NCD	1964.168	−977.0842	1MG-NCD	1098.71	−544.355
	1MG-AD	1966.149	−977.0747	2MG-A	1099.805	−544.902

MG: major genes; MX: major genes plus polygenes; A: additive; AD: additive-dominant; ADI: additive-dominance-epistasis; CD: completely dominance; NCD: negatively CD; EA: equal additive; EAD: equal additive-dominance.

**Table 2:** Candidate genomic intervals for leaf plastochron-related traits.

QTL Label	Chr	Start	End	Interval Length (Mb)	No. of Gene	Reference Genome
qPLA.A02-1	A02	9,045,489	9,478,410	0.43	53	ZS11 v0
qPLA.A02-2	A02	13,517,525	13,661,121	0.14	19	ZS11 v0
qPLA.A04	A04	19,839,469	20,142,478	0.30	35	ZS11 v0
qPLA.A02-1	A02	9,066,916	9,477,102	0.41	46	Darmor-*bzh* V10
qPLA.A02-2	A02	13,609,227	13,728,641	0.12	14	Darmor-*bzh* V10
qPLA.A04	A04	17,128,404	17,432,209	0.30	53	Darmor-*bzh* V10

**Table 3:** Candidate gene information for controlling leaf plastochron in the genomic intervals.

Gene ID	Genomic Position	Best Hit to *A. thaliana*	Function Description
*BnaA02G0156900ZS*	A02:9104462−9107270	*AT1G65480*	Protein FLOWERING LOCUS T
*BnaA02G0160500ZS*	A02:9357771−9367517	*AT1G66350*	DELLA protein RGL1
*BnaA02G0160600ZS*	A02:9375716−9377422	*AT1G56650*	MYB-like, a putative MYB domain-containing transcription factor
*BnaA02G0215300ZS*	A02:13571765−13573401	*AT1G75410*	BLH3, BEL1-like homeodomain protein 3
*BnaA02G0215600ZS*	A02:13592588−13594117	*AT1G47620*	CYP96A8, a member of CYP96A
*BnaA04G0200300ZS*	A04:19944280−19946261	*AT3G44300*	NIT2, nitrilase 2
*BnaA04G0200700ZS*	A04:19964345−19967629	*AT2G30980*	ASK6, a shaggy-related protein kinase
*BnaA04G0201700ZS*	A04:20020034−20020282	*AT2G31081*	CLAVATA3/ESR (CLE)-related protein 4
*BnaA04G0201900ZS*	A04:20029992−20030249	*AT2G31082*	CLAVATA3/ESR (CLE)-related protein 7
*BnaA04G0202000ZS*	A04:20059635−20059880	*AT2G31085*	CLAVATA3/ESR (CLE)-related protein 6

## Data Availability

The raw sequencing data generated in this study are available in SRA (https://www.ncbi.nlm.nih.gov/sra, accessed on 24 January 2025.) of NCBI with the accession numbers PRJNA1055329 and PRJNA1207201.

## Supplementary Materials

The following supporting information can be downloaded at: https://www.mdpi.com/article/10.3390/plants14111719/s1, Figure S1 Venn diagrams of InDels indentifed in the samples; Figure S2 ΔInDel-index calculated based on the ZS11 v0 reference genome; Figure S3 ΔInDel-index calculated based on the Darmor-bzh V10 reference genome; Figure S4 Genome collinearity analyses between the intervals of ZS11 and Darmor-bzh; Figure S5 Principal components analysis (PCA) for the 12 RNA-seq samples based on the candidate gene expression; Figure S6 Boxplots of candidate gene expression and leaf number in the RNA-seq samples; Figure S7 The relationships between flowering time and 24 SNVs shared with natural accessions; Figure S8 Experimental design; Table S1 Statistics of leaf plastochron-related traits in the four-generation population; Table S2 Pearson correlation coefficients among leaf plastochron, primary branch number, biomass, and seed yield; Table S3: Fitness tests of candidate models for ZH18 × 2116 C hybrid combinations; Table S4 Estimates of genetic parameters of leaf plastochron-related traits; Table S5 Statistics of the sequencing data for the four samples; Table S6 Statistical table of candidate genomic intervals on the ZS11 genome; Table S7: Statistics of candidate genomic intervals on the Darmor-bzh genome; Table S8: Results of protein sequence alignment between Darmor-bzh and ZS11; Table S9: Annotation of SNP and InDel in the candidate genes; Table S10: Statistics of variants detected between BSA sequencing and 2311 accessions; Table S11: Candidate gene expression patterns in different tissues of ZS11.
