# Supplementary material for: Genetic Analyses, BSA-Seq, and Transcriptome Analyses Reveal Candidate Genes Controlling Leaf Plastochron in Rapeseed (Brassica napus L.)
Source: Plants (Basel). 2025 Jun 5;14(11):1719. doi: 10.3390/plants14111719 (PMC12156987; doi:10.3390/plants14111719)
Supplement: Supplementary file 1 [file plants-14-01719-s001.zip › FigureS-ok.pptx]

## Slide 1
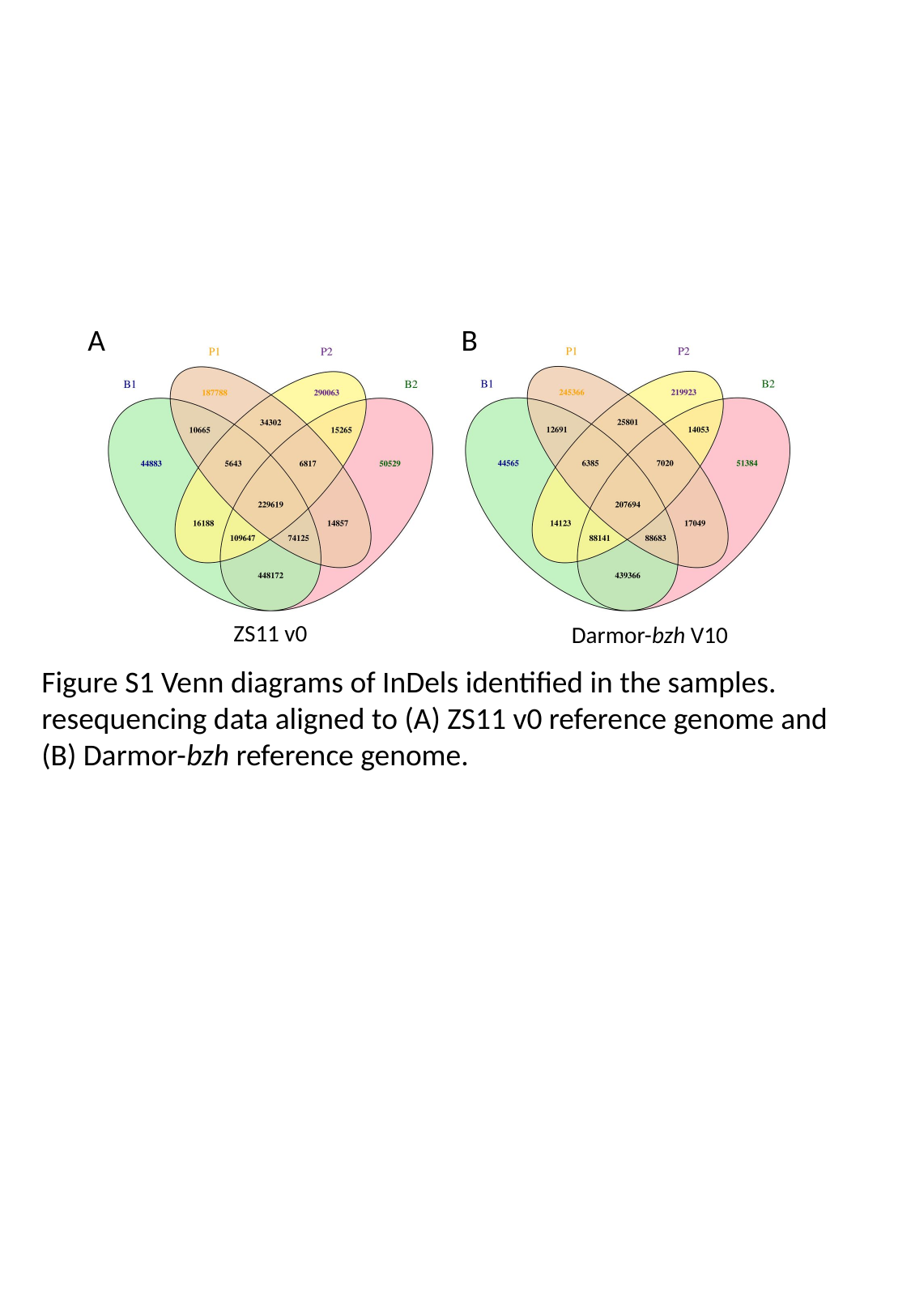

A
B
ZS11 v0
Darmor-bzh V10
Figure S1 Venn diagrams of InDels identified in the samples. resequencing data aligned to (A) ZS11 v0 reference genome and (B) Darmor-bzh reference genome.

## Slide 2
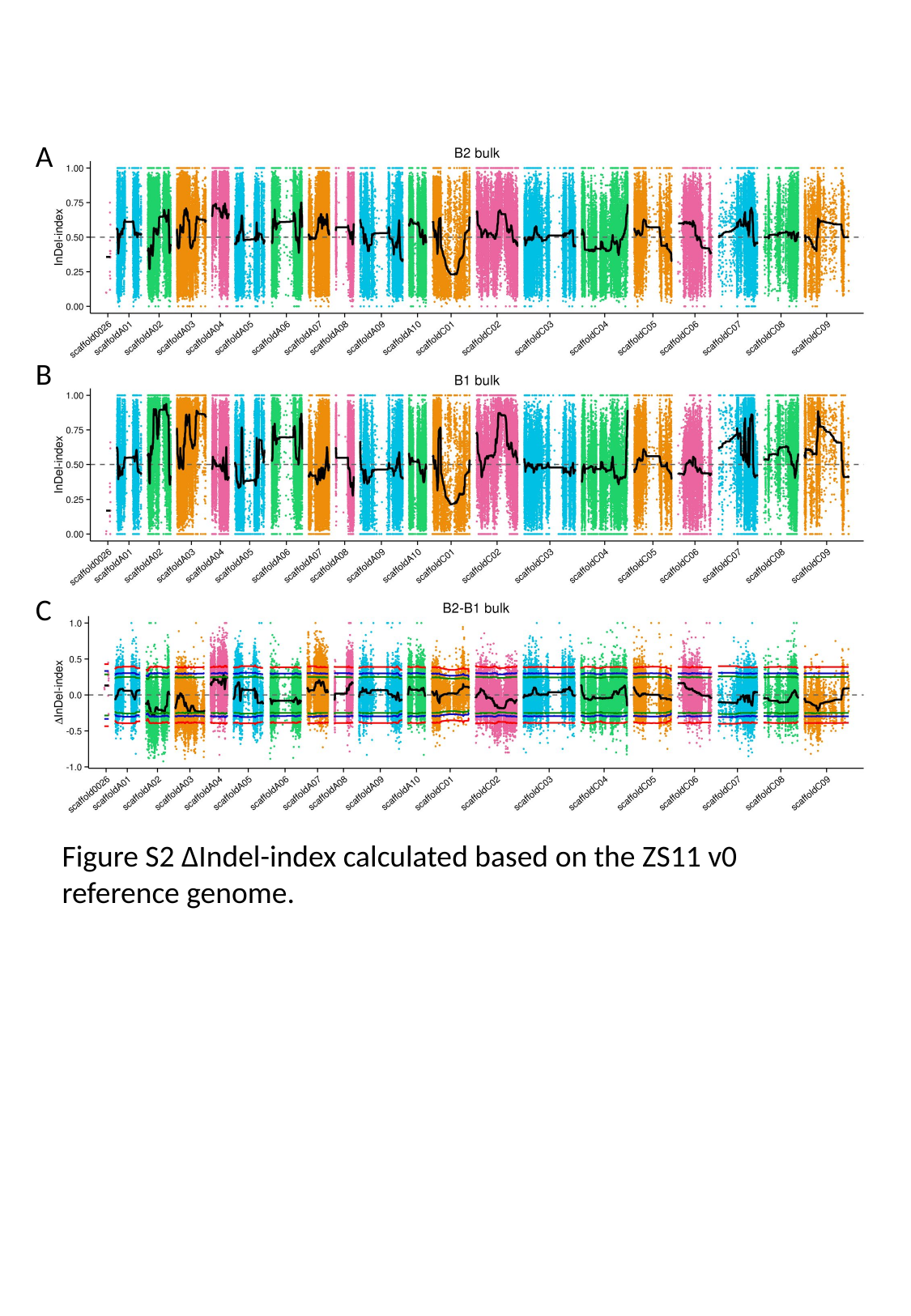

A
B
C
Figure S2 ΔIndel-index calculated based on the ZS11 v0 reference genome.

## Slide 3
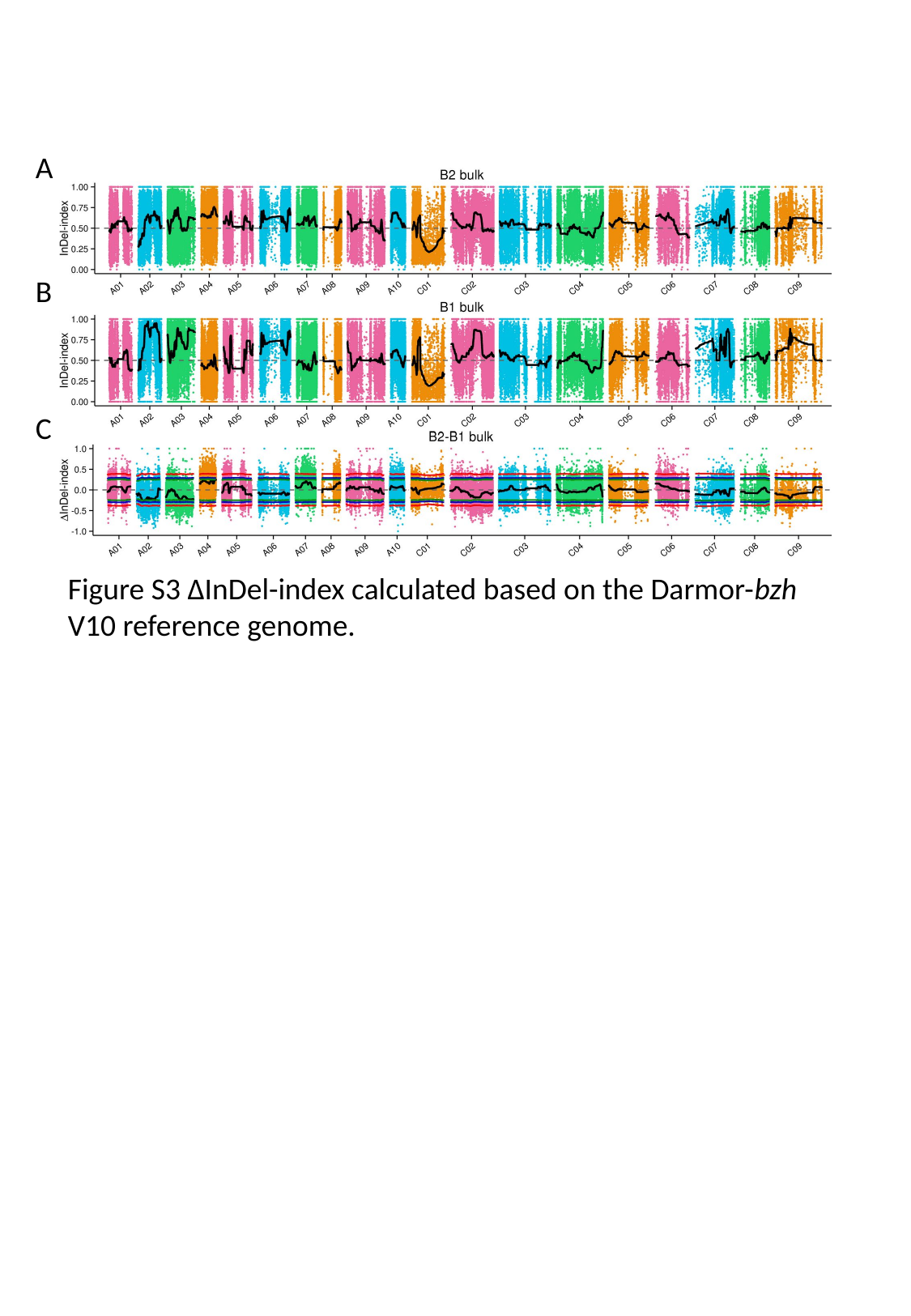

A
B
C
Figure S3 ΔInDel-index calculated based on the Darmor-bzh V10 reference genome.

## Slide 4
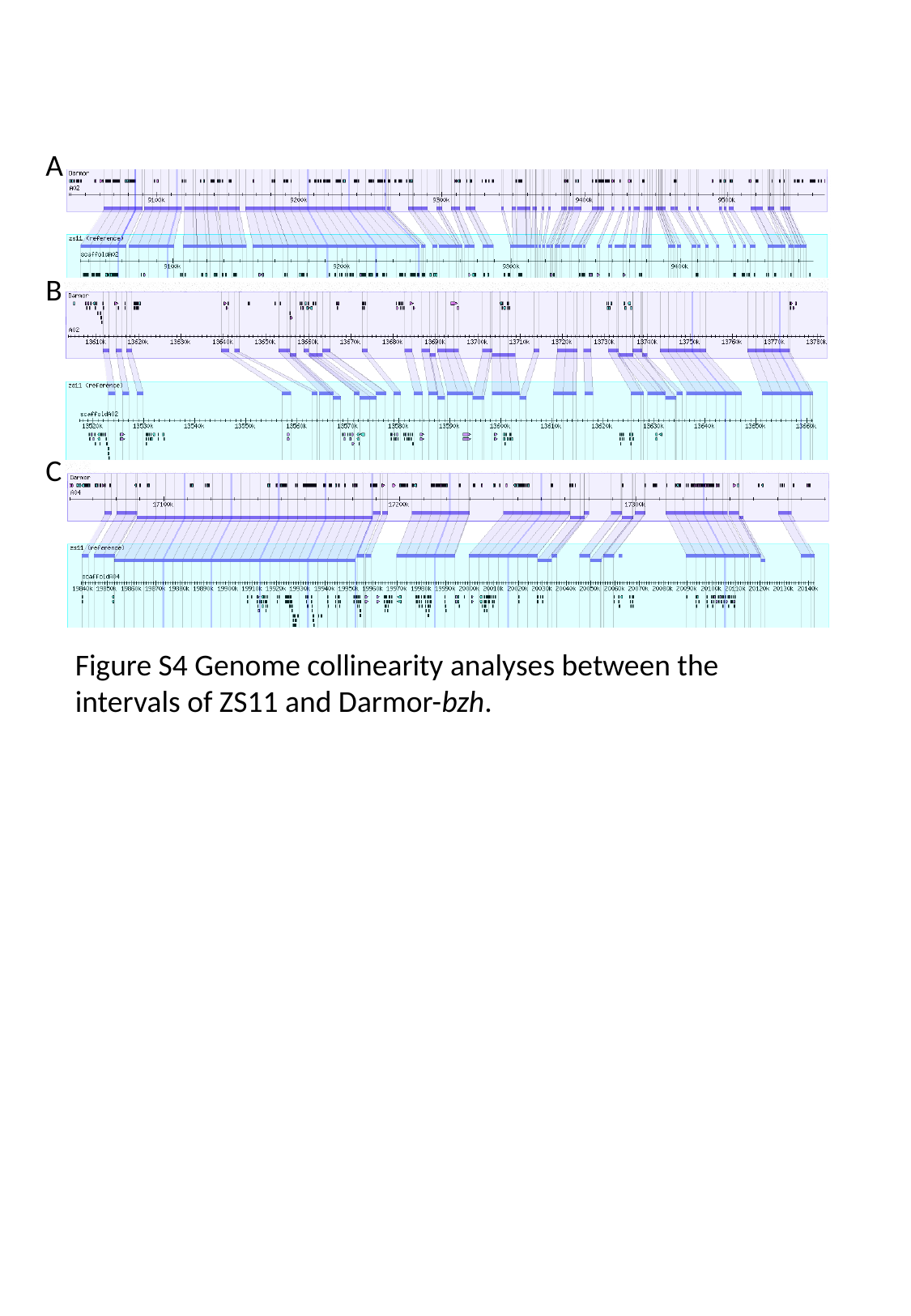

A
B
C
Figure S4 Genome collinearity analyses between the intervals of ZS11 and Darmor-bzh.

## Slide 5
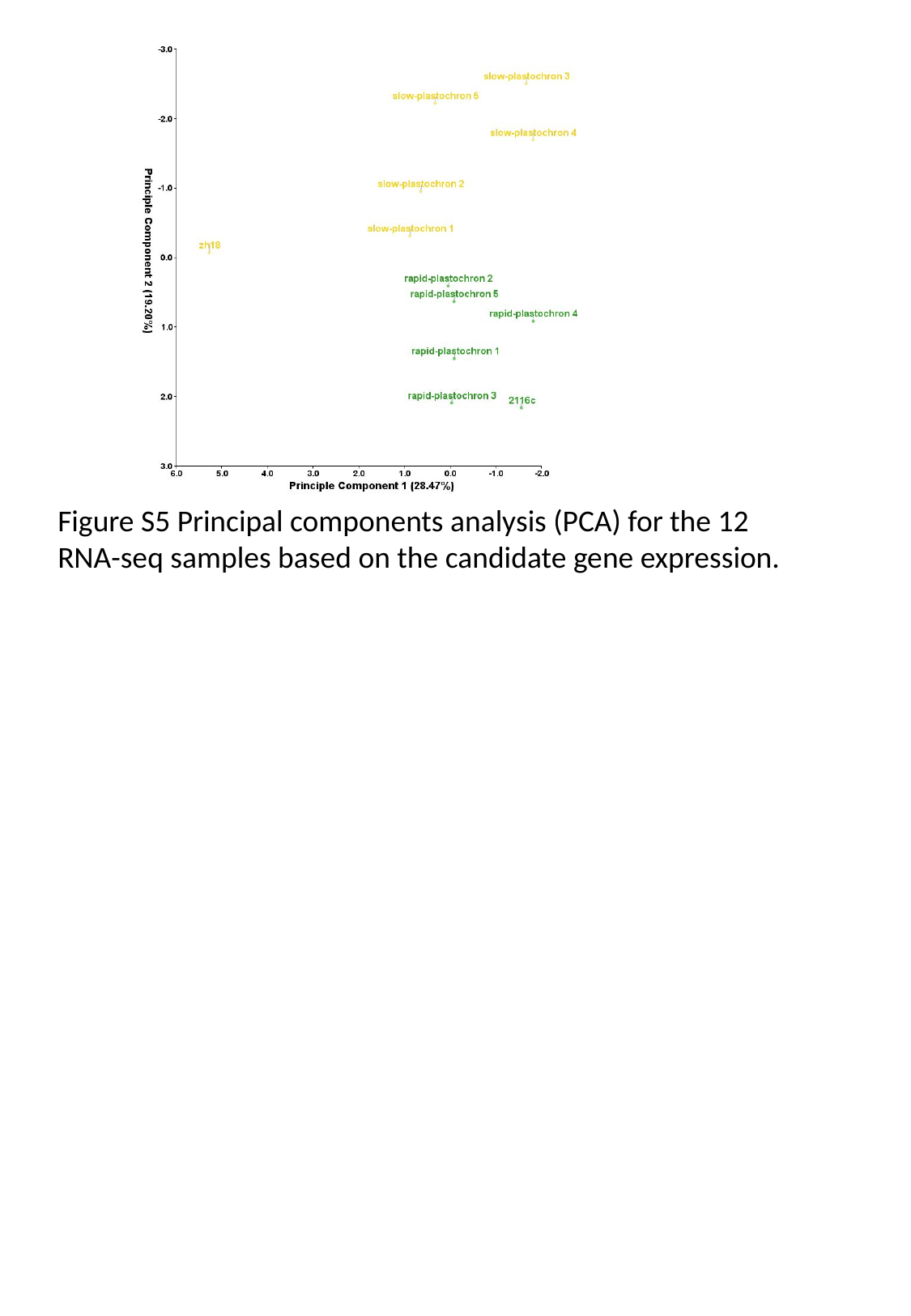

Figure S5 Principal components analysis (PCA) for the 12 RNA-seq samples based on the candidate gene expression.

## Slide 6
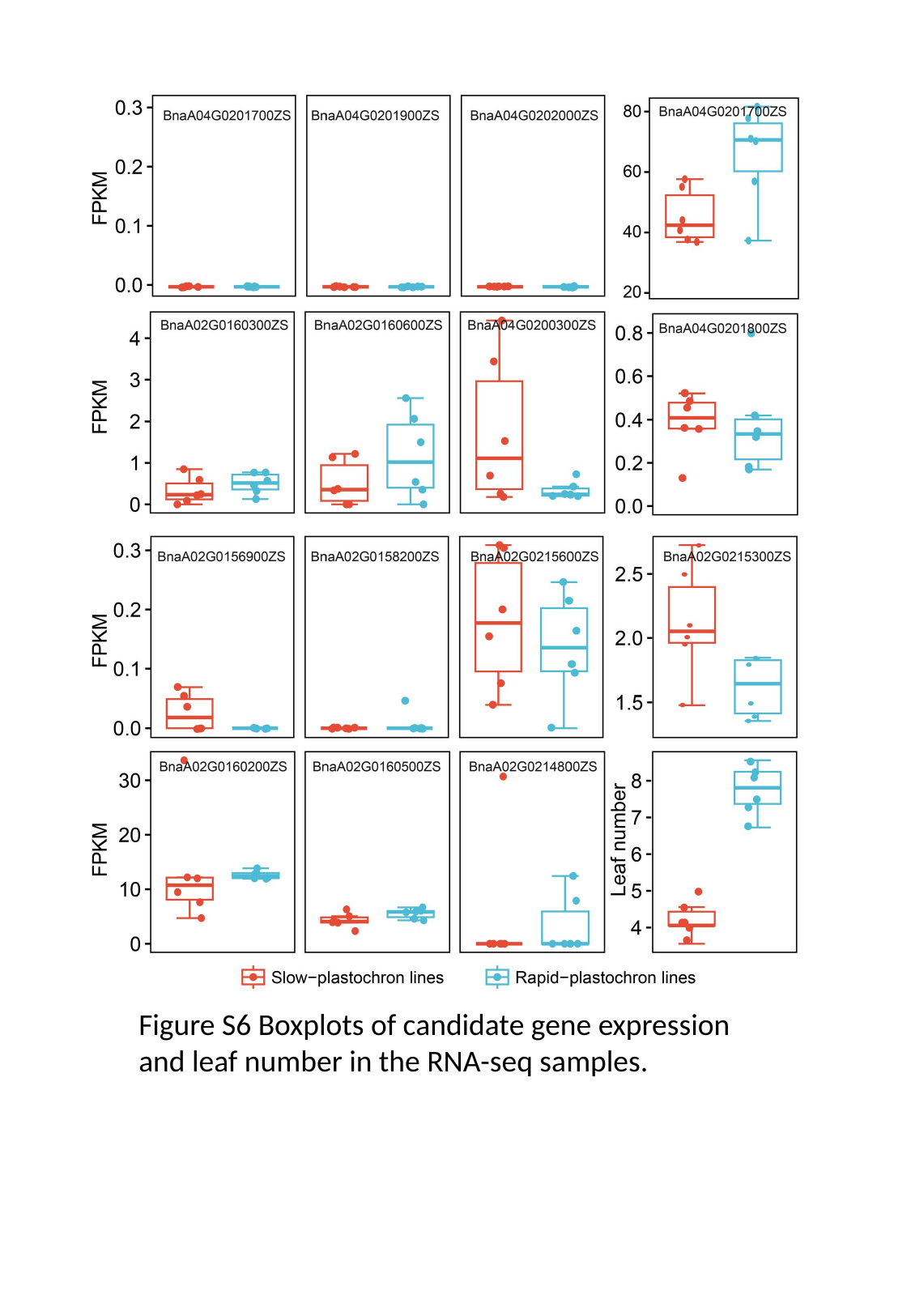

Figure S6 Boxplots of candidate gene expression and leaf number in the RNA-seq samples.

## Slide 7
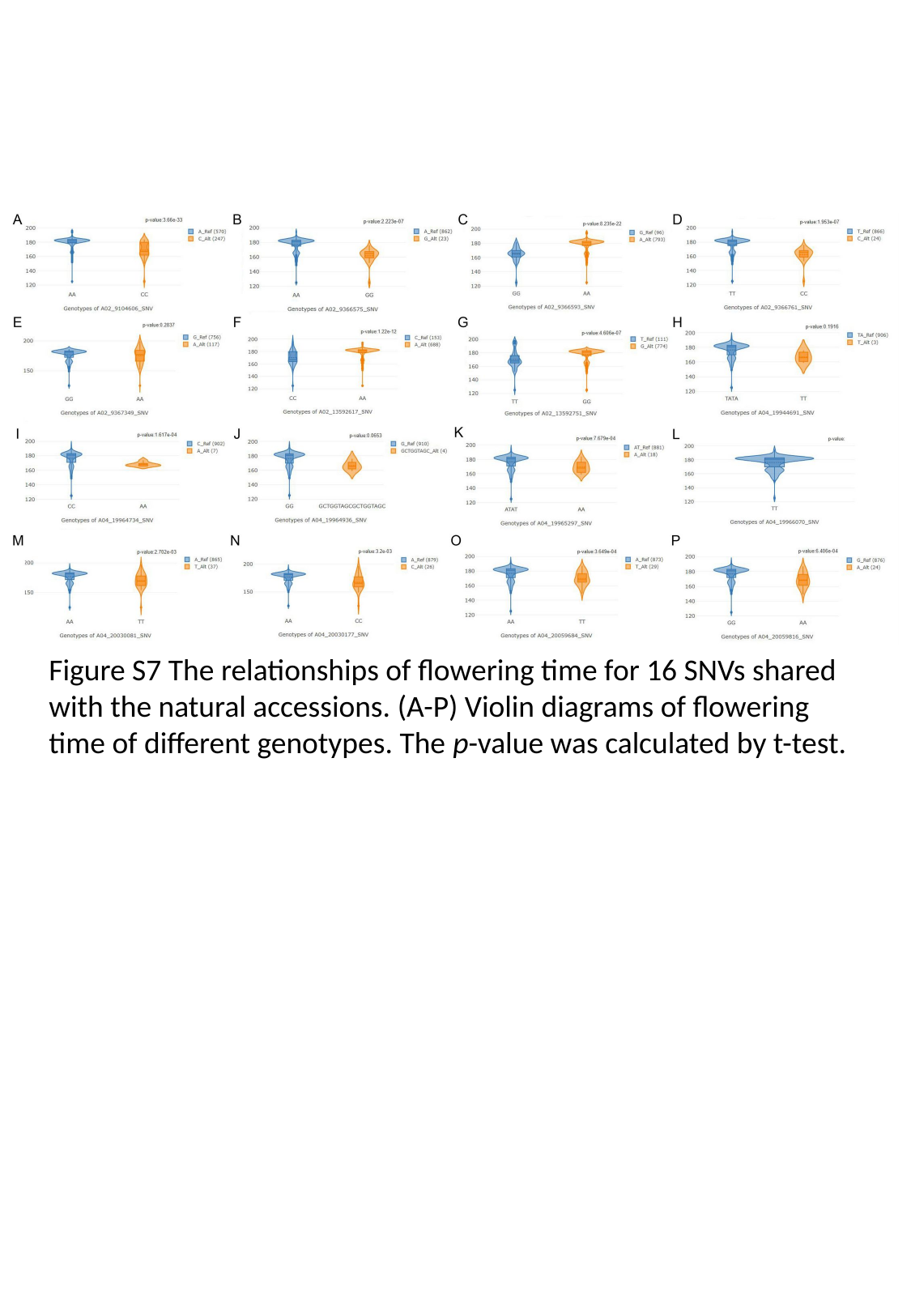

Figure S7 The relationships of flowering time for 16 SNVs shared with the natural accessions. (A-P) Violin diagrams of flowering time of different genotypes. The p-value was calculated by t-test.

## Slide 8
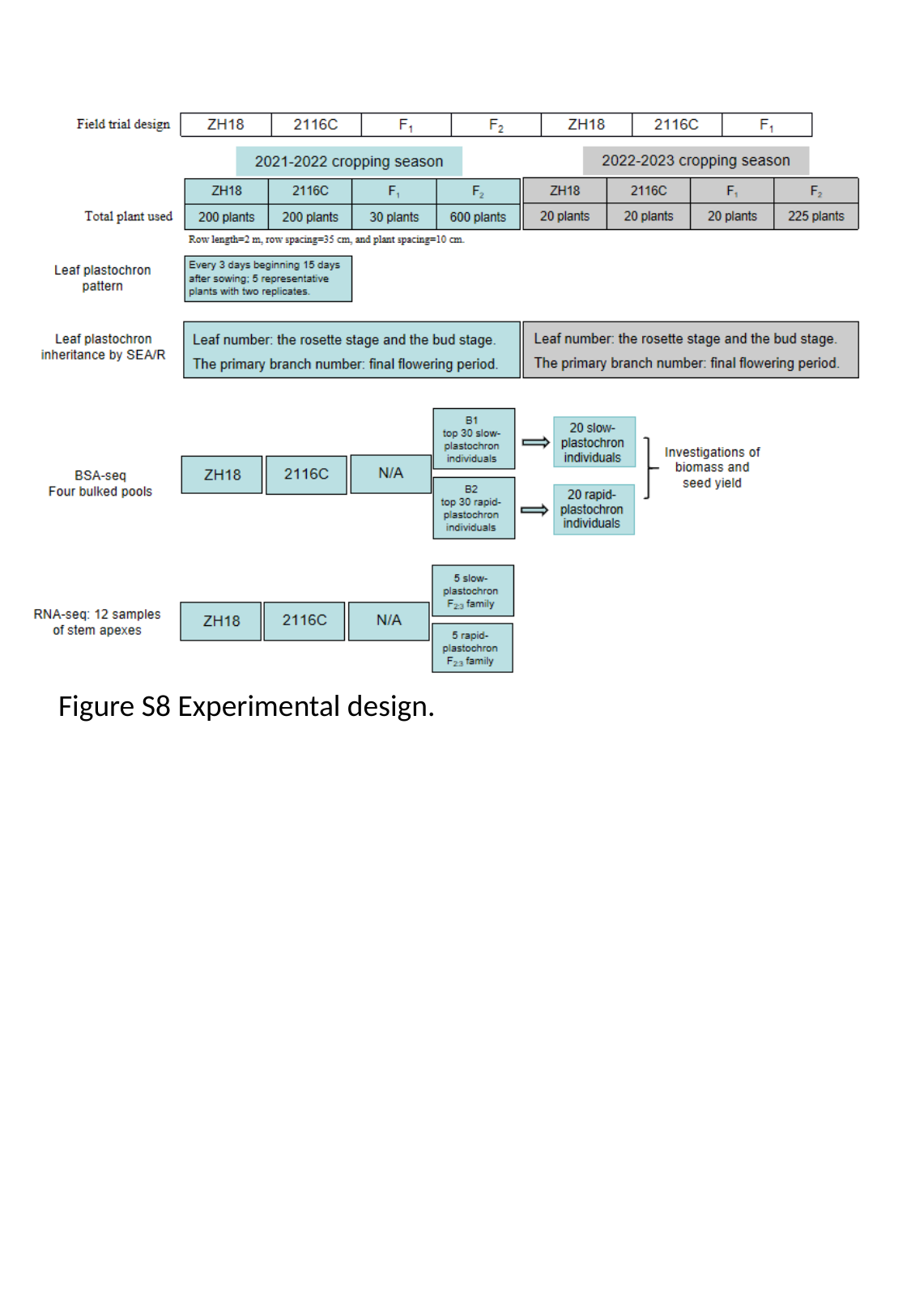

Figure S8 Experimental design.
